# Engineering *Escherichia coli* for production of 4-hydroxymandelic acid using glucose–xylose mixture

**DOI:** 10.1186/s12934-016-0489-4

**Published:** 2016-05-27

**Authors:** Fei-Fei Li, Ying Zhao, Bing-Zhi Li, Jian-Jun Qiao, Guang-Rong Zhao

**Affiliations:** Department of Pharmaceutical Engineering, School of Chemical Engineering and Technology, Tianjin University, Tianjin, 300072 China; Key Laboratory of Systems Bioengineering (Ministry of Education), Tianjin University, Tianjin, China; SynBio Research Platform, Collaborative Innovation Center of Chemical Science and Engineering (Tianjin), Tianjin, China

**Keywords:** 4-Hydroxymandelic acid, *Escherichia coli*, Co-utilization of glucose and xylose, Synthetic biology, Metabolic engineering

## Abstract

**Background:**

4-Hydroxymandelic acid (4-HMA) is a valuable aromatic fine chemical and widely used for production of pharmaceuticals and food additives. 4-HMA is conventionally synthesized by chemical condensation of glyoxylic acid with excessive phenol, and the process is environmentally unfriendly. Microbial cell factory would be an attractive approach for 4-HMA production from renewable and sustainable resources.

**Results:**

In this study, a biosynthetic pathway for 4-HMA production was constructed by heterologously expressing the fully synthetic 4-hydroxymandelic acid synthase (*shmaS)* in our l-tyrosine-overproducing *Escherichia coli* BKT5. The expression level of *shmaS* was optimized to improve 4-HMA production by fine tuning of four promoters of different strength combined with three plasmids of different copy number. Furthermore, two genes *aspC* and *tyrB* in the competitive pathway were deleted to block the formation of byproduct to enhance 4-HMA biosynthesis. The final engineered *E. coli* strain HMA15 utilized glucose and xylose simultaneously and produced 15.8 g/L of 4-HMA by fed-batch fermentation in 60 h.

**Conclusions:**

Metabolically engineered *E. coli* strain for 4-HMA production was designed and constructed, and efficiently co-fermented glucose and xylose, the major components in the hydrolysate mixture of agricultural biomass. Our research provided a promising biomanufacturing route to produce 4-HMA from lignocellulosic biomass.

**Electronic supplementary material:**

The online version of this article (doi:10.1186/s12934-016-0489-4) contains supplementary material, which is available to authorized users.

## Background

4-Hydroxymandelic acid (4-HMA) is widely used in production of aromatic drugs and flavors. It is employed for the preparation of 4-hydroxyphenylacetic acid, which is the synthetic precursor of selective β1-receptor antagonist drug atenolol [1]. 4-HMA can conjugate cytotoxic drug and enzyme substrate, and such a 4-HMA based adaptor system showed promising application in the targeting drug delivery system [2]. Moreover, 4-HMA derivatives, polyhydroxylated mandelic acid amides, were reported with higher radical scavenging activities than that of antioxidants α-tocopherol and butylated hydroxytoluene [3]. Recently, the recombinant *Escherichia coli* converted 3-ethoxy-4-HMA to ethyl vanillin, a widely used flavor in foods, beverages and cosmetics [4].

4-HMA is currently synthesized by condensation of glyoxylic acid with excessive phenol via chemical approach [5], which is facing many challenges. First, feedstock like phenol origins from fossil energy as coal, which is unsustainable. Second, condensation reaction with sodium or potassium hydroxide is extremely environmentally unfriendly, and causes pollution in its production and refinery. Third, subsequent acidification of the reaction and extraction of excessive phenol make downstream process more complicated. At last, such a process produces a mixture including byproduct ortho-isomer 2-hydroxymandelic acid, which is very difficult to separate from 4-HMA. Biotechnological approach would be an attractive alternative for 4-HMA production from renewable and sustainable bioresources.

4-Hydroxyphenylglycine, 4-HMA derivative, is a natural building block for several nonribosomal peptide antibiotics like chloroeremomycin [6] and vancomycin [7]. A gene involved in the biosynthesis of 4-hydroxyphenylglycine was identified to encode 4-hydroxymandelate synthase (HmaS), which catalyzes the conversion of 4-hydroxyphenylpyruvate (4-HPP) to 4-HMA via the oxidative decarboxylation reaction and adds hydroxyl to the side chain rather than the aromatic ring. Natural phenolic products are derived from aromatic amino acid biosynthetic pathway [8], and 4-HPP is the direct precursor of l-tyrosine. In the last few years, notable advances have been achieved in microbial production of 4-HPP derivatives, such as caffeic acid, L-dopa, 4-hydroxystyrene, phenol, salvianic acid A and flavonoid [9–14].

**Fig. 1:**
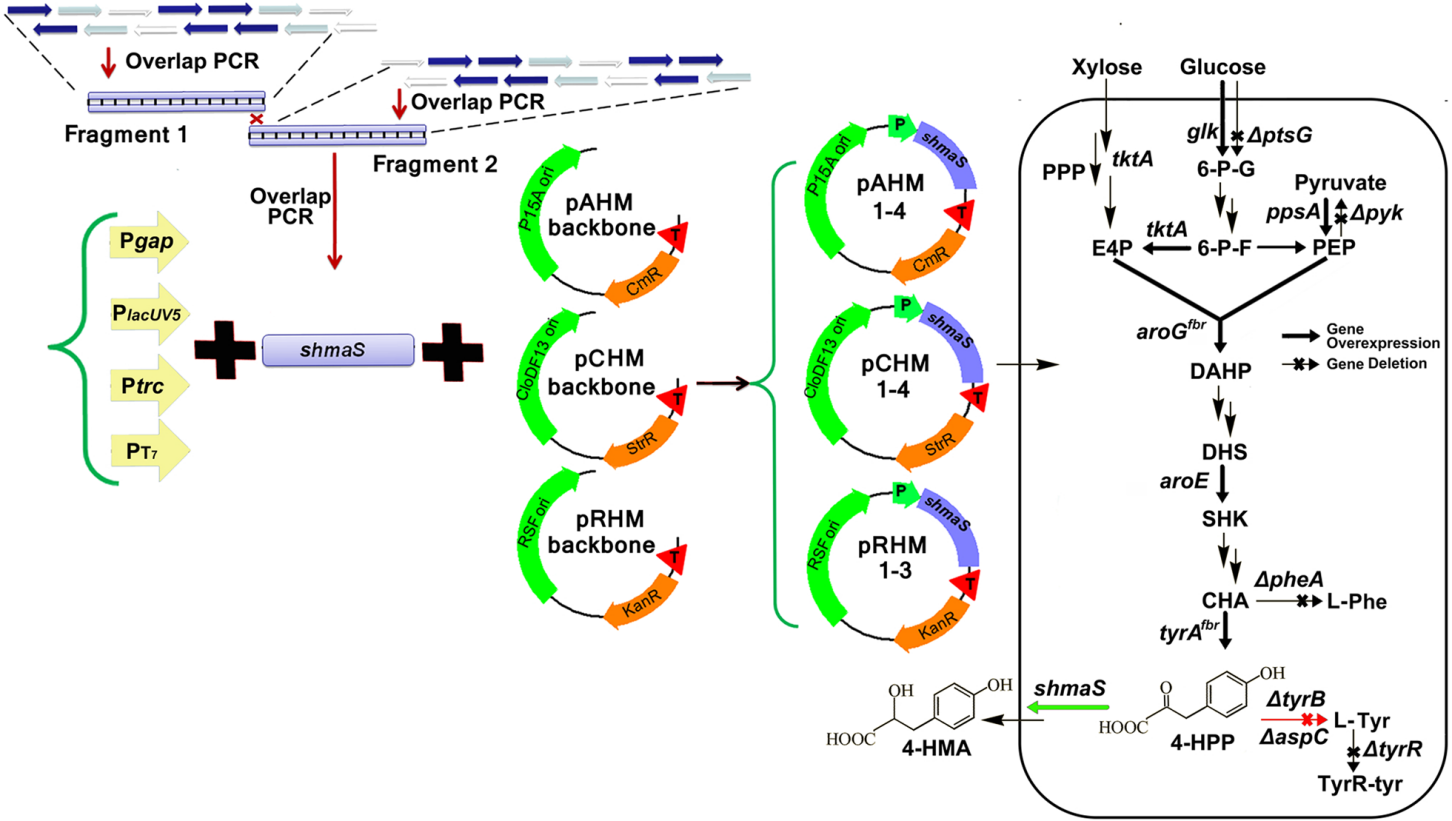
The construction process of recombinant expression vectors and engineered pathway for 4-HMA production. *PPP* pentose phosphate pathway, *6*-*P*-*G* 6-phosphate-glucose, *6*-*P*-*F* 6-phosphate-fructose, *PEP* phosphoenolpyruvate, *E4P* erythrose-4-phosphate, *DAHP* 3-deoxy-D-arabino-heptulosonate-7-phosphate, *DHS* 3-dehydroshikimic acid, *SHK* shikimic acid, *CHA* chorismic acid, *4*-*HPP* 4-hydroxyphenylpyruvic acid, *L*-*Phe* l-phenylalanine; *L*-*Tyr* l-tyrosine;*4*-*HMA* 4-hydroxymandelic acid, *TyrR*-*tyr* TyrR-tyrosine DNA-binding transcriptional repressor

We previously constructed an *E. coli* strain BKT5 capable of overproducing l-tyrosine by deleting five genes (Δ*ptsG*, Δ*pykA*, Δ*pykF*, Δ*tyrR* and Δ*pheA*), and overexpressing *aroG*^*fbr*^-*tyrA*^*fbr*^-*aroE* and *glk*-*tktA*-*ppsA* [13]. In this study, we used BKT5 as the starting strain, and the gene *shmaS* of *Amycolatopsis orientalis* with optimized codons was introduced for 4-HMA production (Fig. 1). By using the strategy of synthetic biology [15] to improve 4-HMA biosynthesis, we finely tuned the expression of synthetic *shmaS* gene by combining promoters (as *gap*, *lacUV5*, *trc* and T7) of various strengths and vectors of various replication origins (as p15A, CloDF13, RSF1030). To further enhance metabolic flux to 4-HMA, we manipulated the genome of *E. coli* BAK5 by deletion of *tyrB* and *aspC*, which would block the competitive pathway from 4-HPP to formation of l-tyrosine. The final engineered *E. coli* strain HMA15 was employed to produce 4-HMA using glucose and xylose by fed-batch fermentation. To our knowledge, this is the first report describing engineered *E. coli* for producing 4-HMA using glucose–xylose mixture.

## Methods

### Strains, plasmids, primers and reagents

Strains, plasmids and primers used in this study are listed in Tables 1, 2 and 3, respectively. *E. coli* BW25113 derivatives were used to construct 4-HMA producing strains. All chemical reagents were purchased from Sigma Aldrich (Beijing, China). Plasmids were isolated using the Tianprep Mini plasmid Kit purchased from Tiangen (Beijing, China). DNA Gel Extraction Kit from Tiangen was used to isolate DNA fragments from agarose gels. All PCR fragments were validated via DNA sequencing provided by BGI. Oligonucleotides and synthetic long DNA fragments were ordered from GenScript (Nanjing, China). DNA polymerase Fastpfu and Taq for PCR was purchased from TransGen Biotech (Beijing, China). All restriction enzymes and rapid DNA ligase were purchased from Thermo Scientific (Beijing, China). *E. coli* DH5α competent cells were used for the propagation of recombinant plasmids.

**Table 1:** Strains used in this study

Strains	Characteristics	Source
BAK5	*E. coli* BW25113 *∆ptsG, ∆tyrR, ∆pykA, pykF, ∆pheA*	[13]
BAK6	BAK5 *∆tyrB*	[13]
BAK7	BAK6 *∆aspC*	This study
BKT5	BAK5 with pYBT5	[13]
BKT6	BAK6 with pYBT5	This study
BKT7	BAK7 with pYBT5	This study
HMA	*E. coli* BL21(DE3) with pHMA	This study
HMA01	BKT5 with pAHM1	This study
HMA02	BKT5 with pAHM2	This study
HMA03	BKT5 with pAHM3	This study
HMA04	BKT5 with pAHM4 and pYBH1	This study
HMA05	BKT5 with pCHM1	This study
HMA06	BKT5 with pCHM2	This study
HMA07	BKT5 with pCHM3	This study
HMA08	BKT5 with pCHM4 and pYBH1	This study
HMA09	BKT5 with pRHM1	This study
HMA10	BKT5 with pRHM2	This study
HMA11	BKT5 with pRHM3	This study
HMA12	BKT6 with pCHM3	This study
HMA13	BKT6 with pRHM1	This study
HMA14	BKT6 with pRHM3	This study
HMA15	BKT7 with pCHM3	This study
HMA16	BKT7 with pRHM1	This study
HMA17	BKT7 with pRHM3	This study

**Table 2:** Plasmids used in this study

Plasmids	Characteristics	Source
pBldgbrick1	pMB1 ori with P_lacUV5_ and P_trc_; Ap^R^	[13]
pYBT5	pBldgbrick 1 with P_*lacUV5*_ *aroG* ^*fbr*^ *tyrA* ^*fbr*^ *aroE,* P_*trc*_ *ppsA tktA glk*	[13]
pYBH1	pYSC1 with P_*lacUV5*_ T7 RNA polymerase	[13]
pEBM	pEASY-Blunt with *shmaS*; pUC ori; Kan^R^	This study
pHMA	pET28a with *shmaS*	This study
pACYCDuet-1	p15A ori; Cm^R^	Novagen
pCDFDuet-1	CloDF13 ori; Str^R^	Novagen
pRSFDuet-1	RSF1030 ori; Kan^R^	Novagen
pAHM	p15A ori; Cm^R^	This study
pCHM	CloDF13 ori; Str^R^	This study
pRHM	RSF1030 ori; Kan^R^	This study
pAHM1	pAHM with P_gap_-*shmaS*	This study
pAHM2	pAHM with P_lacUV5_-*shmaS*	This study
pAHM3	pAHM with P_trc_-*shmaS*	This study
pAHM4	pACYCDuet-1 with P_T7_-*shmaS*	This study
pCHM1	pCHM with P_gap_-*shmaS*	This study
pCHM2	pCHM with P_lacUV5_-*shmaS*	This study
pCHM3	pCHM with P_trc_-*shmaS*	This study
pCHM4	pCDFDuet-1 with P_T7_- *shmaS*	This study
pRHM1	pRHM with P_gap_-*shmaS*	This study
pRHM2	pRHM with P_lacUV5_-*shmaS*	This study
pRHM3	pRHM with P_trc_-*shmaS*	This study

**Table 3:** Primers used in this study

Name	Sequence (5′–3′)
gap F1	CCATGGTTTAGGAGGATTACAAAATGCAGAACTTCGAAATCGACTACG
gap R1	CGGGATCCCTAACGACGTGCCGCACCGA
gap F2	CCCAAGCTTGCGTAATGCTTAGGCACA
gap R2	ACTGCTTGTTCTTGTGGCGCCATATATTCCACCAGCTATT
lacUV5F1	GGACTAGTATGCAGAACTTCGAAATCGACTACG
lacUV5R1	CGGGATCCCTAACGACGTGCCGCACCGA
lacUV5F2	CGGGATCCGCGCCCAATACGCAAACCG
lacUV5R2	CCCAAGCTTCTAACGACGTGCCGCACCGA
trcF1	GGAATTCCATATGCAGAACTTCGAAATCGACTACG
trc F2	CGGGATCCCTGCAGCGACTGCACGGTG
T7 F	GGAATTCCATATGCAGAACTTCGAAATCGACTACG
T7 R	GGGGTACCCTAACGACGTGCCGCACCGA
aspC F	CGGACTTCCCTTCTGTAACCATAATGGAACCTCGTCATGATG
aspC R	GTGTAGGCTGGAGCTGCTTC
aspC VF	AGCCCGCTTTTCAGCGGGCTTCATTGTTTTTAATGCTTACATG
aspC VR	GGAATTAGCCATGGTCC
aspC YZF	CCTGCGTTTTCATCAGTAATAGTTGG
aspC YZR	CCTTATCCGGCCTACAAAATCG
	GGCGAAGAAGTTGTCCATA
	TTGAGGCATTTCAGTCAGT

### Codon optimization and assembly of synthetic *hmaS* gene

Starting from amino acid sequence of HmaS (GenBank ID CAA11761.1) of *Amycolatopsis orientalis*, we optimized the codon usage for *hmaS* heterologous expression in *E. coli* using a design procedure *JCat*, and Shine–Dalgarno-like ribosomal pause sequences and selected restriction enzyme recognition sites were also removed. Then, the full DNA sequence of *hmaS* was divided into twenty-six oligonucleotides for each one about 60 bp long with 20 bp region homologous to its adjacent oligonucleotides. The full length of the *hmaS* gene was divided into two fragments and assembled by two-step overlapping PCR. The 5′-terminal and 3′-terminal fragments of the *hmaS* gene optimized were assembled by first-step overlapping PCR with the first fourteen and the latter fourteen oligonucleotides, respectively. PCR reaction system contained fourteen oligonucleotides each with 0.2 μM, 0.2 mM dNTPs, 2.5 units TransFast pfu DNA polymerase, 1× TransFast pfu buffer in the final volume of 50 μL, and 30 cycles of 95 °C for 5 s, 55 °C for 15 s, 72 °C for 15 s were carried out for PCR amplification program. Then the full-length of the *hmaS* gene was amplified by the second-step overlapping PCR using these two overlapped fragments with the first and last oligonucleotide primers. Synthetic *hmaS* gene determined to be correct by sequencing was designated as *shmaS*, and cloned into vector pEASYBlunt, resulting pEBM. The *shmaS* gene was cloned into expression vector pET28a under control of T7 promoter, resulting pHMA.

### Combinatorial construction of shmaS-regulated expression vectors

In order to screen superior promoter-copy number combinations for 4-HMA producing, four promoters P_*gap*_, P_*trc*_, P_*lacUV5*_ and P_T7_ with various expression strength and three types of vectors with various copy number (p15A, CloDF13, and RSF1030 as replicon) were selected, and eleven expression vectors were constructed as shown in Table 2. The skeleton vectors pAHM, pCHM and pRHM without *lacI* were obtained by PCR with pACYCDuet-1, pCDFDuet-1 and pRSFDuet-1 as templates, respectively.

For the construction of pAHM1, pCHM1 and pRHM1, the *shmaS* gene was amplified from pEBM by PCR with primers gapF1 and gapR1. Promoter *gap* was cloned from genome of *E. coli* BW25113 with primers gapF2 and gapR2. The *shmaS* gene and promoter *gap* were spliced by overlapping extension PCR, constructing P_*gap*_-*shmaS*, which was then ligated at the restriction sites *Hin*dIII and *Bam*HI of skeleton vectors pAHM, pCHM and pRHM, respectively. For the construction of pAHM2, pCHM2 and pRHM2, the *shmaS* gene was amplified from pEBM with primers lacUV5F1 and lacUV5R1, which was then cloned into pBldgBrick1 obtaining recombinant, and with it as template, lacUV5F2 and lacUV5R2 as primers yielding P_*lacUV5*_-*shmaS*, which was then cloned into the skeleton vectors pAHM, pCHM and pRHM, respectively. The same method was used for construction of pAHM3, pCHM3 and pRHM3. For the construction of pAHM4 and pCHM4, the *shmaS* gene was amplified from pEBM by PCR with primers T7F and T7R, which was then cloned into the restriction sites *Nde*I and *Kpn*I of pACYCDuet-1 and pCDFDuet-1, respectively, under the regulation of T7 promoter directly. Due to the same antibiotic resistance of pRHM and pYBH (for expression of T7 RNA polymerase), the expression vector pRHM4 with combination of P_T7_-*shmaS* and the skeleton vector pRHM was not constructed.

### Genome modification and construction of recombinant strains

*E. coli* BAK5 was used as the starting strain, in which five genes involved in glucose transportation and l-tyrosine biosynthetic pathway had been deleted [13]. Strain BAK6 was constructed in our previous study [13]. Strain BAK7 was constructed by further deletion of the *aspC* gene in strain BAK6 using the classical λ-red recombination method [16]. Template pKD3 and primers aspCF and aspCR were used to clone the fragment for replacing the *aspC* gene with a chloramphenicol resistant selectable marker. Primers aspCVF and aspCVR were used to verify the positive knock-out strain and aspCYZF and aspCYZR to verify the final selectable marker removed strain. Stains BKT5, BKT6 and BKT7 were constructed by transformation of pYBT5 into strain BAK5, BAK6 and BAK7, respectively. Then transformation of different recombinant plasmids constructed in Table 2 into strains BKT5, BKT6 or BKT7 contributed to *shmaS* heterologous expression strains as characterized in Table 1.

### Fermentation media and cultivation conditions

LB medium was used for cultivating the general strains for cloning or preparation of fermentation seeds. The fermentation of engineered *E. coli* strain was operated at 37 °C and 220 rpm with 50 mL medium in 250 mL shake flasks with synthetic minimal salt medium. The salt medium contains 17.1 g/L Na_2_HPO_4_·12H_2_O, 3.0 g/L KH_2_PO_4_, 0.5 g/L NaCl, 3.0 g/L NH_4_Cl, 0.2 g/L l-phenylalanine, 3.0 g/L l-aspartic acid, 1.0 g/L yeast extract, 1.0 g/L MgSO_4_·7H_2_O, 0.03 g/L CaCl_2_·2H_2_O, 0.02 g/L FeSO_4_·7H_2_O. The initial medium pH was adjusted to 7.0 by the addition of 10 M NaOH. Sole glucose (4.0 g/L), xylose (4.0 g/L), or mixture of glucose (2.0 g/L) and xylose (2.0 g/L) were added as carbon source supply. For *shmaS* expression controlled by promoter T7, the preliminary experiments showed that IPTG induction inhibited cell growth, and *shmaS* was expressed without additional inducer IPTG in the fermentation process. For *shmaS* expressions controlled by the promoters *gap*, *trc* and *lacUV5*, they were constitutively expressed throughout the fermentation.

For bioreactor fermentation, the 5 L fermenter (Bailun, Shanghai) was employed, and contained 2 L the same medium as described in shake flask except for initial 5.0 g/L glucose, 5.0 g/L xylose and 5.0 g/L yeast extract. 300 ml seed culture was inoculated into the bioreactor, yielding an initial OD_600_ of ~0.3. The dissolved oxygen (DO) level was maintained at 30 % (v/v) by flowing 2 L/min of air with automatically changing the agitation speed from 300 to 700 rpm. The feeding solution contained 250 g/L glucose, 250 g/L xylose, 60 g/L yeast extract, 3.0 g/L l-aspartate, 0.2 g/L l-phenylalanine. The speed of feeding was regulated to keep the concentration of residual sugars at appropriate level. Fed-batch fermentation was performed twice and average data were shown.

Broth samples were withdrawn periodically to determine the cell biomass, concentrations of residual sugars and metabolites produced. Each fermentation was conducted in triplicates. Antibiotics were supplemented to the media when needed at the following concentrations: 50 mg/L ampicillin, 30 mg/L kanamycin, 30 mg/L streptomycin, 20 mg/L chloramphenicol.

### Analytical methods

Cell growth was monitored by measuring the absorbance at 600 nm (OD_600_) using an UV–VIS spectrophotometer. Glucose consumption was quantified by a biosensor SBA-90 (Biology Institute of Shandong Academy of Sciences, China). Residual concentration of xylose was measured using Waters 1515 HPLC system, equipped with a Bio-Rad HPX-87H column and a refractive index detector (Waters 2414, Milford, USA), and the column was eluted at 65 °C with 5 mM sulfuric acid at 0.6 mL/min.

The broth samples were centrifuged, and supernatants were filtered through 0.22 μm syringe filter, and injected to the HPLC system. 4-HMA and l-tyrosine were measured using Agilent 1200 HPLC system equipped with a C18 column (250 × 4.6 mm, Agilent) and a PDA detector (Agilent) at 196 nm with a mobile phase (10 % methanol-90 % H_2_O, addition of 0.1 % formic acid) at 1.0 mL/min. The structure of 4-HMA was identified using LC–MS (Agilent 1200 HPLC system and 6310 Ion Trap mass spectrum system, Agilent) under negative ion mode. All of the HPLC analysis were quantified using a six point standard curve and the *R*^*2*^ coefficient for the standard curve was higher than 0.99.

## Results and discussion

### Expression of synthetic *shmaS* gene with optimized codons led to 4-HMA production

The *hmaS* gene encodes an α-keto acid-dependent dioxygenase in the biosynthesis of polycyclic nonribosomal glycopeptide antibiotic chloroeremomycin [6, 7]. It acts on phenyl α-keto acid to decarboxylate, and one oxygen from molecular oxygen is incorporated into the carbonyl group and one into the benzylic hydroxyl group to form α-hydroxyl acid [17]. HmaS can catalyze the conversion of 4-HPP and phenylpyruvate to 4-HMA and mandelate, respectively, and the biosynthetic pathways for production of D-/L-phenylglycine, and S-/R-mandelic acid from phenylpyruvate were constructed in *E. coli* [18–20]. Enzymatic activity of *A. orientalis* HmaS towards 4-hydroxyphenylpyruvate (4-HPP) was higher than that of *S. coelicolor* HmaS [18]. Thus, we chose *hmaS* from actinomyces *A. orientalis* to construct a heterologous 4-HMA biosynthetic pathway in *E. coli.*

Native *hmaS* sequence of *A. orientalis* is characterized with high GC content (over 72 %) and not suitable to express in *E. coli*. Optimization of the coding sequence of *hmaS* is necessary for the efficient heterologous expression. According to codon usage bias of *E. coli*, and elimination of the secondary structure of its mRNA, we designed the full length DNA sequence of *hmaS* and designated as *shmaS*. Full sequence of *shmaS* was conveniently assembled by overlapping PCR (detailed description in “Methods” section). The expression vector pHMA was constructed and introduced to strain BL21 (DE3), resulting the recombinant strain HMA.

**Fig. 2:**
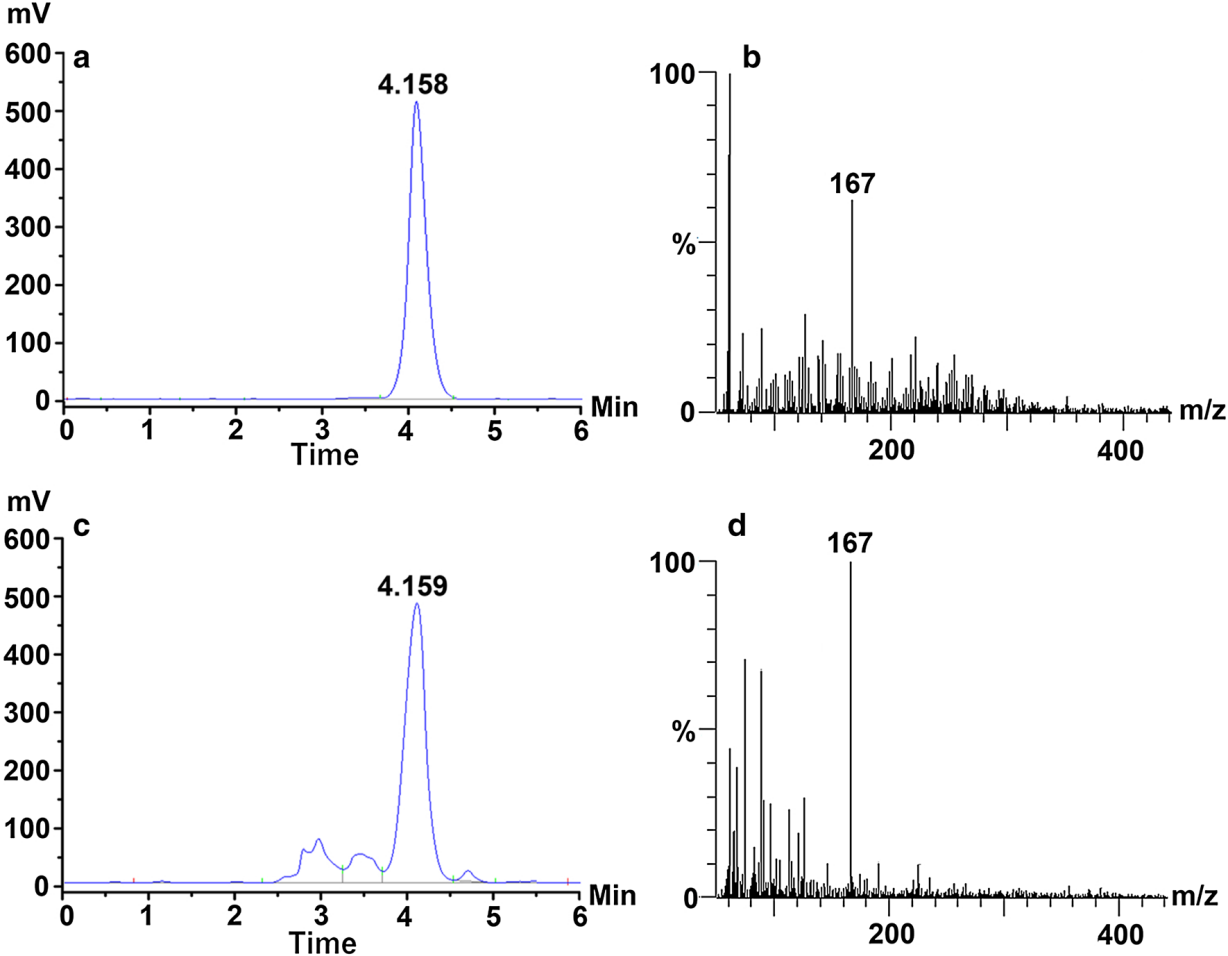
HPLC and LC–MS analysis of 4-HMA produced by *shmaS*-expressing strain HMA. **a** HPLC analysis of standard 4-HMA. **b** LC–MS analysis of standard 4-HMA. **c** Identity of 4-HMA detected in the fermentation supernatant by HPLC analysis, compared the retention time to standard 4-HMA. **d** The identity of 4-HMA detected in the fermentation supernatant by LC–MS analysis, compared m/z to standard 4-HMA

Strain HMA was cultivated in mineral salt medium with addition of 0.4 % glucose and 0.5 g/L l-tyrosine. After 24 h of cultivation, the supernatant of fermentation broth was analyzed by HPLC, and 4-HMA was identified by LC–MS analysis. Strain HMA carrying *shmaS* produced a major product that has the same retention time as standard 4-HMA (Fig. 2a, c) and the identical [M-H]^−^ ions to standard 4-HMA (Fig. 2b, d). The primary ion fragment at m/z 167 ([M-H]^−^) (Fig. 2d) corresponds to 4-HMA with molecular weight of 168. These results confirm the functionality of optimized and synthetic *shmaS* gene in the biosynthesis of 4-HMA via its expression in *E. coli.*

We aimed at expanding the aromatic amino acid pathway to establish a novel biosynthetic platform for the microbial production of 4-HMA. HmaS of the 4-hydroxyphenylglycine biosynthetic pathway in *A. orientalis* was characterized to convert 4-HPP to 4-HMA [6] by the unusual catalytic mechanism of decarboxylation and dioxidation [7]. l-tyrosine biosynthetic pathway provides precursor 4-HPP [21], which would be converted to 4-HMA by heterologous expression of synthetic *shmaS* in this study (Fig. 1). Thus we constructed the new metabolic pathway for the production of 4-HMA in *E. coli*.

### Fine tuning of *shmaS* expression improved 4-HMA production by combination of promoters and copy numbers

The expression levels of genes involved in the pathway affect the production of enzymes encoded and corresponding products, but the precise artificial regulation of a specific gene in a specific host context is hard to achieve [22]. Thus strategies trying to tune gene expression have been developed, among them, promoter engineering [23], and fine tuning by combinatorial regulation of promoter strength and copy number [24] displayed efficiency for metabolic engineering and synthetic biology. Here, we regulated the expression of *shmaS* gene by combining four promoters of various strength (as P_*gap*_, P_*lacUV5*_, P_*trc*_, P_T7_) and three plasmids with various replication origins (as p15A, CloDF13, RSF1030).

**Fig. 3:**
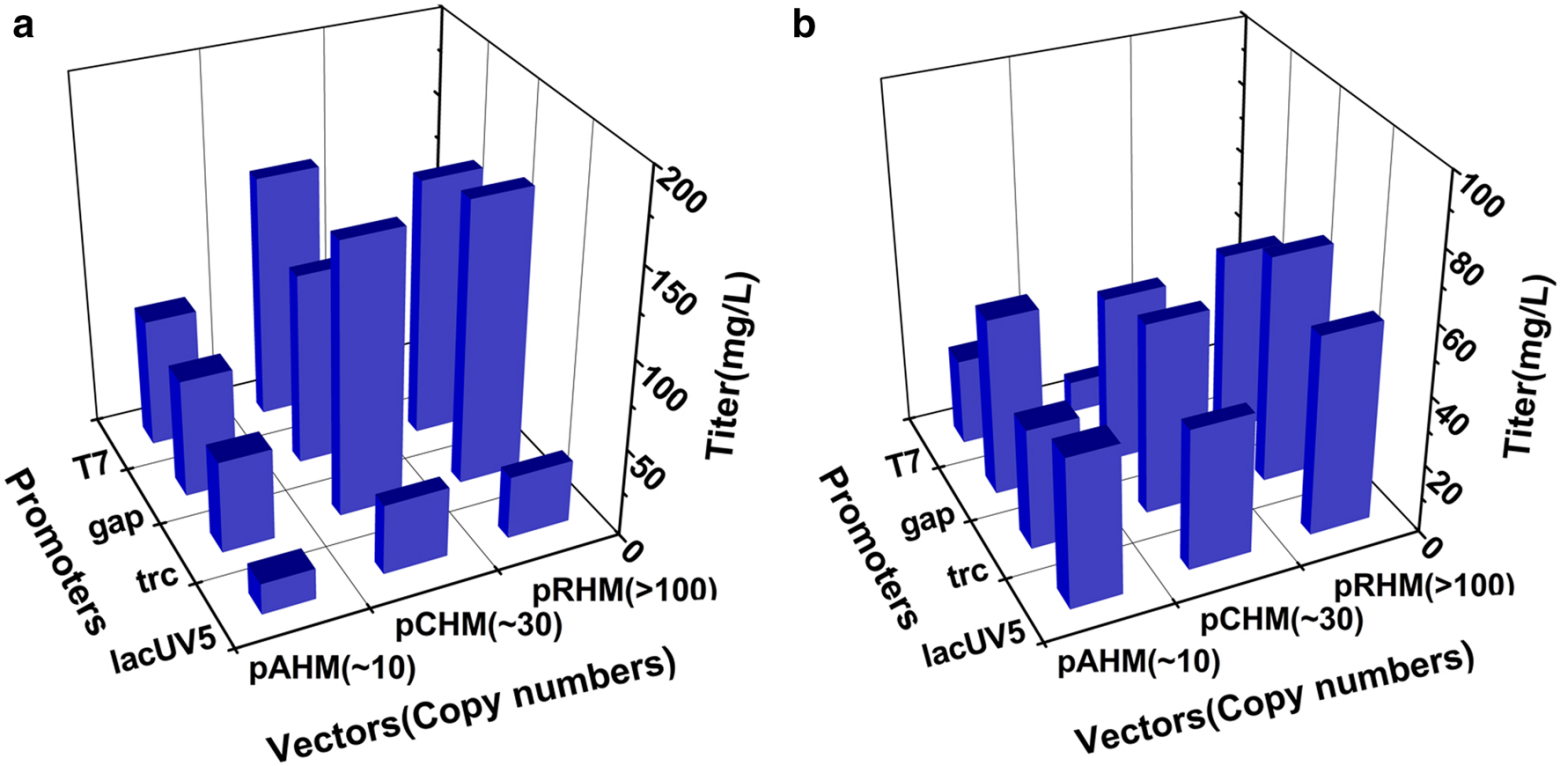
Fine tuning of *shmaS* expression by combinatorial regulation for screening 4-HMA high-yielding strains at 24 h of fermentation. **a** 4-HMA production. **b** l-tyrosine accumulation

All eleven recombinant *E. coli* strains expressing *shmaS* on various vectors produced 4-HMA, and their titers were largely diverse (Fig. 3a, Additional file 1: Table S1). After 24 h of fermentation, strain HMA02 produced 4-HMA with the lowest titer of 17.15 mg/L, while HMA11 produced 4-HMA with the highest titer of 160.05 mg/L, and the ratio of the highest to the lowest 4-HMA titer was 9.3-fold. Obviously, the fine tuning strategy of combinatorial regulation in expression of *shmaS* helped us screen out three higher yielding strains HMA07, HMA09 and HMA11, the titers of 4-HMA were 153.59, 146.68 and 160.05 mg/L, respectively.

Among three types of plasmids, copy numbers with replication origin p15A, CloDF13 and RSF1030 are estimated about 10, 30 and 100, respectively [25]. When weak promoter *lacUV5* drove expression of *shmaS* on the low or high copy number vectors, the lowest titer of 4-HMA was produced, compared with promoters T7, *trc* and *gap*. Hybrid promoter *trc* is popularly used in metabolic engineering [26] and its strength is considered to be stronger than that of *lacUV5*. When the *shmaS* was expressed under the control of promoter *trc*, the titers of 4-HMA were approximately three-fold of increase, compared to promoter *lacUV5* on middle and high copy-number vectors. Promoter T7 is well known as a very strong promoter, and widely used in production of heterologous proteins and metabolic engineering. Herein, the expression of *shmaS* on the middle copy-number vector under control of promoter T7 could be beneficial for production of 4-HMA, but not optimal. Promoter *gap* is a strong constitutive promoter regulating expression of glyceraldehyde 3-phosphate dehydrogenase in glycolysis pathway, and ever been used for high production of resveratrol [27]. The expression of *shmaS* driven by the *gap* promoter on high copy-number vector resulted in the production of 4-HMA up to 146.68 mg/L in strain HMA09, however promoter *gap* was lesser efficient to express *shmaS* for 4-HMA production, compared with promoter *trc* on the same vector.

Although fine tuning of *shmaS* expression through combination of promoters and gene copy-numbers improved the production of 4-HMA in l-tyrosine overproducing background strains by using glucose, as shown in Fig. 3b (Additional file 1: Table S1), byproduct l-tyrosine was unexpectedly accumulated in fermentation broth. HmaS can also convert phenylpyruvate to mandelic acid [18–20], however, no phenylpyruvate and its derivative byproducts were detectable in our *pheA* deficient strains. Incomplete conversion of metabolic intermediate 4-HPP to 4-HMA would lead to accumulation of l-tyrosine (Fig. 1). It indicated that metabolic flux to l-tyrosine could compete the formation of 4-HMA. We speculated that blocking the flux from 4-HPP to l-tyrosine might further improve the production of 4-HMA.

### Genome modification enhanced 4-HMA production by blocking byproduct formation

In order to enhance the production of 4-HMA, a logical possibility can be considered by blocking the conversion of 4-HPP to l-tyrosine through modifying the genome of strain BAK5, which was constructed by deleting *ptsG, tyrR, pheA, pykA, and pykF* in our previous study [13]. In *E. coli,* two aminotransferases are involved in the last step of the biosynthesis of l-tyrosine [28]. Under normal conditions, the aromatic amino acid aminotransferase encoded by *tyrB* is the main enzyme for l-tyrosine biosynthesis from 4-HPP. In case of higher 4-HPP pool, the aspartate aminotransferase encoded by *aspC* could contribute to the biosynthesis of l-tyrosine as well. In order to verify our hypothesis, sequentially inactivating the *tyrB* and *aspC* genes from the l-tyrosine biosynthetic pathway was implemented. We deleted *tyrB* and *aspC* in strain BAK5 background in sequence by λ-red recombinant method and constructed two new strains BAK6 and BAK7 for expression of *shmaS*. After introduction of pYBT5 (Table 2) and three *shmaS* expression vectors (pCHM3, pRHM1 and pRHM3), resulting engineered strains were cultivated for fermentation 4-HMA. 3.0 g/L of L-aspartate was added in mineral salt medium to support growth of strains with *aspC* deletion.

**Fig. 4:**
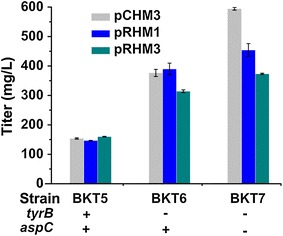
Titers of 4-HMA produced by genome modification through deleting *tyrB* and *aspC* at 24 h fermentation

As expected, for *tyrB* or/and *aspC* deletion strains, byproduct l-tyrosine was totally undetectable, meanwhile an obvious increase in 4-HMA production was observed (Fig. 4), in comparison with the unmodified strains. Deletion of *tyrB* led to 1.45-fold increase of 4-HMA titer of 376.26 mg/L in strain HMA12 (pCHM3) at 24 h fermentation. Further deletion of *aspC* contributed to improvement of 4-HMA production, but the efficacy depended on the expression vectors of *shmaS.* Compared to expression vectors pRHM1 and pRHM3, vector pCHM3 gave the highest titer of 4-HMA production in strain BKT7 background with double deletions of *tyrB* and *aspC*. Strain HMA15 (pCHM3) produced 594.07 mg/L of 4-HMA, 30.8 and 59.2 % higher than strains HMA16 (pRHM1) and HMA17 (pRHM3), respectively.

TyrB and AspC are identified as isoenzymes [29] and also involved in biosynthesis of l-phenylalanine [30]. In previous reports, *tyrB* and *aspC* were deleted to block the biosynthesis of l-phenylalanine, and titers of phenylpyruvate [31] and its derivatives R-/S-mandelic acid [19], d-/l-phenylglycine [18, 20] and benzyl alcohol [32] were greatly improved. In this study, deletion of *tyrB* and *aspC* completely blocked the formation of byproduct l-tyrosine from precursor 4-HPP and resulted in significant increase of 4-HMA production. Therefore, double deletions of *tyrB* and *aspC* could benefit for the fermentative production of aromatic 4-HMA from 4-HPP and its analogs, mandelic acid, phenylglycine and benzyl alcohol from phenylpyruvate.

### Fed-batch fermentation for production of 4-HMA by co-utilization of xylose and glucose

**Fig. 5:**
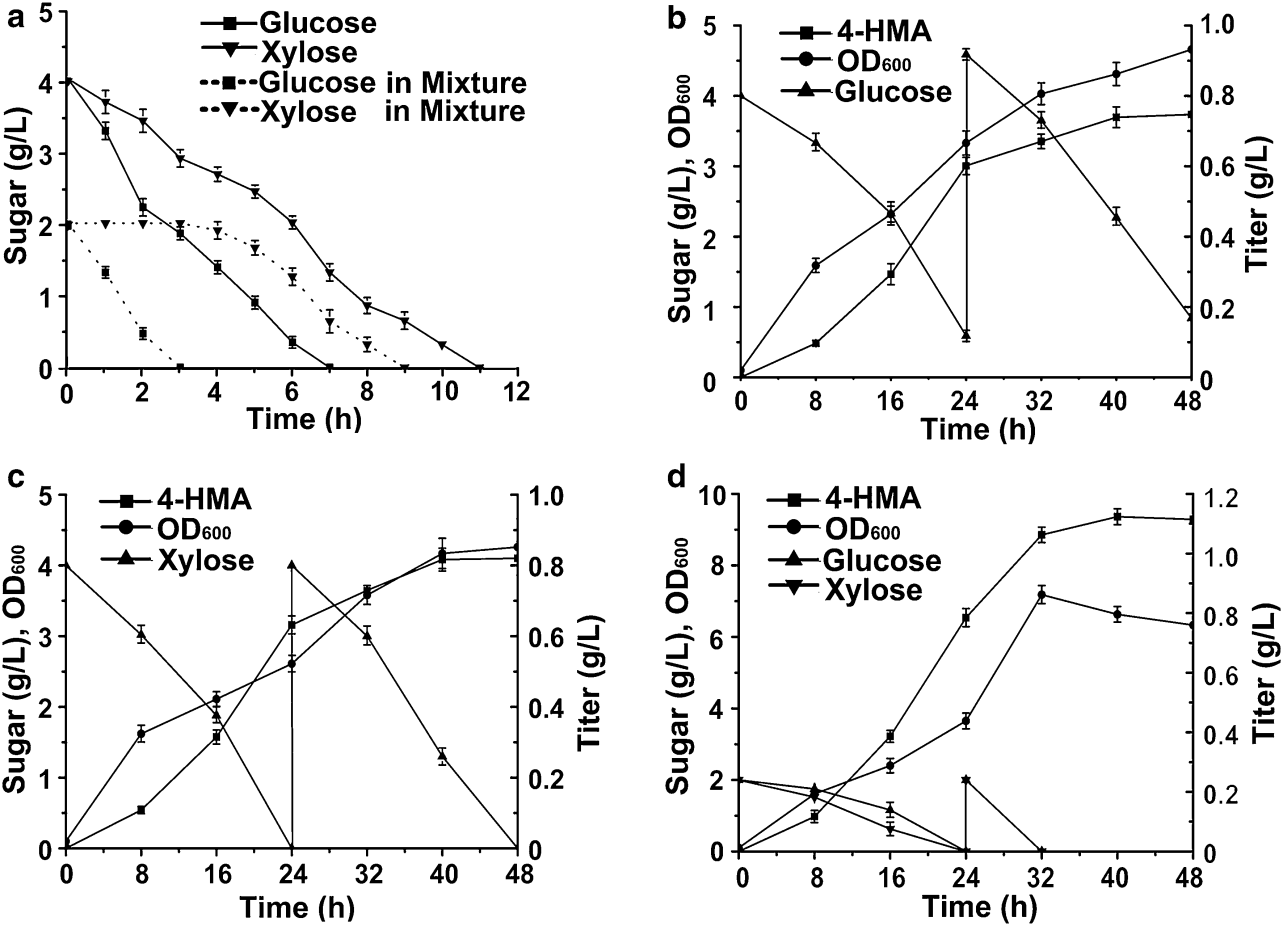
4-HMA production, cell growth and sugar consumption in shake flasks. **a** Consumption of sole sugar and sugars mixture of the wild type *E. coli* BW25113. **b** Fed-batch fermentation of HMA15 with sole glucose as carbon source. **c** Fed-batch fermentation of HMA15 with sole xylose as carbon source. **d** Fed-batch fermentation of HMA15 with mixture of glucose and xylose as carbon sources

Lignocellulosic biomass is the most abundant bioresource in nature, utilization of lignocellulose hydrolysate in fermentation industry for the production of fine chemicals and biofuels is very promising [33]. However, simultaneous utilization of glucose and xylose in lignocellulose hydrolysate is still a challenge, because *E. coli* metabolizes glucose in preference to other sugars [34]. As shown in Fig. 5a, carbon catabolite repression (CCR) by glucose was obvious in the wild type *E. coli* BW25113, and sequential consumption of carbon sources was evident. When cultivated with glucose or xylose as the sole carbon source, the wild type BW25113 consumed glucose faster than xylose. However, when glucose and xylose mixture was provided, the wild type BW25113 consumed glucose rapidly while xylose was used after glucose was significantly depleted.

To release glucose repression, phosphoenolpyruvate-carbohydrate phosphotransferase system (PTS) was often focused as an important target of genetic modification [35]. The *ptsG* gene is responsible for transportation of glucose into cytoplasm and deleted in strain HMA15 (Table 1). Thus fermentation of 4-HMA in *ptsG* deficient strain HMA15 was comparatively carried out in shake flasks by three fed-batch modes, glucose or/and xylose used as sole or mixed carbon source. As shown in Fig. 5b, when glucose was used as sole carbon source, strain HMA15 produced 601.56 mg/L of 4-HMA at 24 h fermentation. With additional amount of glucose fed, the titer of 4-HMA was continuingly increased to 747.13 mg/L at 48 h. When sole xylose was fed as carbon source, strain HMA15 produced 820.18 mg/L of 4-HMA, slightly higher than fed with glucose at 48 h (Fig. 5c). Furthermore, Fermentation was carried out by feeding glucose–xylose mixture. Different ratios of glucose and xylose as carbon source showed that sugar mixture of glucose (2.0 g/L) and xylose (2.0 g/L) at equal amount resulted in the highest titer of 4-HMA at 48 h fermentation (Additional file 1: Figure S1). When equal mass amounts of both glucose and xylose were fed as mixed carbon source, strain HMA15 produced 1.11 g/L of 4-HMA (Fig. 5d), displaying 48.6 and 35.3 % higher than that fed with glucose and xylose, respectively.

**Fig. 6:**
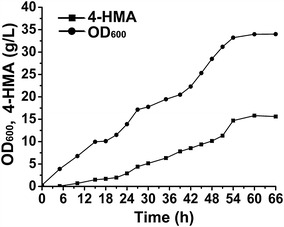
Fed-batch fermentation of HMA15 with glucose–xylose mixture in 5 L bioreactor

Feeding fermentation of strain HMA15 was further performed for production of 4-HMA in 5 L bioreactor. Feeding solution was added into the bioreactor based on concentration of residual sugars to maintain it lower than 2.0 g/L. In all, 42.5 g/L glucose and 42.5 g/L xylose were consumed in bioreactor fermentation. The production of 4-HMA showed a cell growth-dependent manner (Fig. 6). The maximum cell density (OD_600_) was ~34.0 at 60 h, and 4-HMA titer was 15.8 g/L with a productivity of 0.26 g/L h^−1^. During the fermentation, byproduct acetate (3.4 g/L) was detected. It might be caused by the overflux of sugar metabolism. Reduction of acetate accumulation [36] would further improve the yield and titer of 4-HMA.

*E. coli* utilizes xylose as a secondary sugar by the general control of CCR. PTS is the most efficient system for transporting sugars, and plays a crucial role in CCR. PEP-dependent glucose-specific PTS is composed of four components including the soluble non-sugar-specific enzymes EI and HPr (encoded by *ptsI* and *ptsH*, respectively), and glucose-specific soluble enzyme EIIA (encoded by *crr*) and membrane-integral permease EIICB (encoded by *ptsG*). Inactivating one of the PTS components could abolish CCR in *E. coli*, and the effects of different CCR-insensitive mutants on cell growth and sugar consumption were varied, which showed different application for production of various products [37]. Here we showed that *ptsG* negative strain HMA15 simultaneously utilized glucose and xylose (Fig. 5d). It was consistent to previous studies when equal mass of glucose–xylose mixture was employed [38, 39]. In *E. coli* without *ptsG*, GalP and MglABC are involved in glucose transport, but they are less effective than PtsG [40]. In addition, *pykF*- and *pykA*-deficiency in strain HMA15 might lead to slow glucose metabolism as corresponding pyruvate kinase catalyze formation of pyruvate and release energy ATP for glucose phosphorylation and cell growth. When the CCR was abolished, xylose could be transported via non-PTS XylE and XylFGH [34] and catabolized via pentose phosphate pathway (PPP). Furthermore, overexpression of *tktA* coding transketolase A in strain HMA15 would enhanced xylose metabolism and benefit to the formation of precursor E4P for aromatic compound production [41]. Meanwhile, inactivation of *ptsG*, *pykF* and *pykA* in strain HMA15 would increase carbon flux from glucose to PEP via glycolysis, which is precursor for aromatic compound biosynthesis (Fig. 1). Taking together, co-utilization of glucose and xylose would further promote the production of aromatic 4-HMA in strain HMA15.

Recently, several alternative strategies have been proposed to achieve co-utilization of glucose and xylose in *E. coli*. Constitutive expression of xylose metabolism was required from the *araC* mutant strain by adaptive evolution in xylose minimal medium, which showed the simultaneous co-utilization of glucose and xylose and possessed the same growth pattern as the wild type [39]. Similarly, growth adaptation of *ptsG* deficient *E. coli* strain on arabinose resulted in the simultaneous utilization of glucose, xylose and arabinose [42] and was used to produce 3-hydroxybutyric acid [43]. By using SIMUP algorithm, growth phenotype of *E. coli* was predicted; co-utilization of glucose and xylose was constructed by deleting *pgi*, *rpe* and *eda* [44]. It is the first report to achieve co-utilization of two sugars without targeting the regulatory pathways of CCR. Protein engineering of the hexose- or pentose- specific transporters might be orthogonal strategy to confer the co-utilization of glucose and xylose [45, 46].

## Conclusions

Biosynthesis of 4-HMA was achieved through heterologous expression of fully synthetic *shmaS* gene in *E. coli*. Expression of *shmaS* was optimized to improve production of 4-HMA by combinatorial regulation with various promoters and copy numbers. Production of 4-HMA was further increased by deletion of *tyrB* and *aspC* on *E. coli* genome to block the formation of byproduct l-tyrosine. Furthermore, fed-batch fermentation mode with both sugars showed that engineered strain can utilize xylose and glucose simultaneously, and glucose–xylose mixture gave high production of 4-HMA with the titer of 15.8 g/L at 60 h fermentation in 5 L bioreactor. It is expected that 4-HMA production would be further improved through control of acetate biosynthesis and optimization of the fermentation process. Co-utilization of glucose and xylose will be promising for the production of other aromatic products of interest.

## Additional file

10.1186/s12934-016-0489-4 Fine tuning of *shmaS* expression by combinatorial regulation of promoters and copy numbers. **Figure S1.** Production of 4-HMA with different ratio of glucose and xylose.

## Abbreviations

CCR: carbon catabolite repression
E4P: erythrose-4-phosphate
4-HMA: 4-hydroxymandelic acid
HmaS: 4-hydroxymandelate synthase
4-HPP: 4-hydroxyphenylpyruvic acid
PEP: phosphoenolpyruvate
PPP: pentose phosphate pathway
PTS: phosphotransferase system
