# Supplementary material for: Engineering Escherichia coli for production of 4-hydroxymandelic acid using glucose–xylose mixture
Source: Microb Cell Fact. 2016 May 27;15:90. doi: 10.1186/s12934-016-0489-4 (PMC4884394; doi:10.1186/s12934-016-0489-4)
Supplement: Supplementary file 1 — 10.1186/s12934-016-0489-4 Fine tuning of shmaS expression by combinatorial regulation of promoters and copy numbers. Figure S1. Production of 4-HMA with different ratio of glucose and xylose. [file 12934_2016_489_MOESM1_ESM.docx]

Table S1 Fine tuning of *shmaS* expression by combinatorial regulation of promoters and copy numbers for screening 4-HMA high-yielding strains

| Strains | Vectors  (copy numbers) | Promoters | 4-HMA  (mg/L) | L-Tyrosine  (mg/L) |
| --- | --- | --- | --- | --- |
| HMA01 | pAHM (~10) | gap | 66.59 ± 1.88 | 51.09 ± 1.68 |
| HMA02 | pAHM (~10) | lacUV5 | 17.15 ± 1.66 | 43.13 ± 1.26 |
| HMA03 | pAHM (~10) | trc | 51.7 ± 2.42 | 34.75 ± 1.15 |
| HMA04 | pAHM (~10) | T7 | 72.58 ± 2.47 | 24.82 ± 0.98 |
| HMA05 | pCHM(~30) | gap | 108.88 ± 3.61 | 48.45 ± 3.57 |
| HMA06 | pCHM(~30) | lacUV5 | 39.34 ± 1.36 | 40.62 ± 2.81 |
| HMA07 | pCHM(~30) | trc | 153.59 ± 6.98 | 54.95 ± 1.00 |
| HMA08 | pCHM(~30) | T7 | 138.55 ± 6.09 | 8.9 ± 0.86 |
| HMA09 | pRHM(>100) | gap | 146.68 ± 5.4 | 53.11 ± 2.13 |
| HMA10 | pRHM(>100) | lacUV5 | 35.15 ± 2.13 | 57.39 ± 4.01 |
| HMA11 | pRHM(>100) | trc | 160.05 ± 6.24 | 65.55 ± 3.33 |





Figure S1 Production of 4-HMA with different ratio of glucose and xylose
